# Pulse pressure associates with severity and worse outcomes in patients with stable coronary artery disease

**DOI:** 10.1038/s44325-026-00118-5

**Published:** 2026-05-04

**Authors:** Yan Zhang, Sha Li, Hui-Wen Zhang, Yuan-Lin Guo, Na-Qiong Wu, Cheng-Gang Zhu, Rui-Xia Xu, Jie Qian, Ke-Fei Dou, Jian-Jun Li

**Affiliations:** Cardiometabolic Center, State Key Laboratory of Cardiovascular Disease, FuWai Hospital, National Center for Cardiovascular Diseases, Chinese Academy of Medical Sciences, Peking Union Medical College, Beijing, China

**Keywords:** Cardiology, Diseases

## Abstract

The association of pulse pressure (PP) with the severity and cardiovascular (CV) outcomes in stable coronary artery disease populations with normal ejection fraction are sparse. We enrolled 7,027 patients and follow-up 36.4 months, a total of 289 events occurred. Both systolic blood pressure (SBP) and PP were significantly increased by Gensini Score quartiles. Meanwhile, the percentage of 3-diseased vessels was increasing by PP quartiles (*p* < 0.001). On univariate analysis, both PP and SBP were associated with CV death, stroke, and combined outcome (*p* < 0.05, all). However, after adjusting for potential confounders, PP remains significantly associated with stroke (HR 95%CI 1.019[1.005–1.033]) and combined outcome (HR 95%CI 1.014[1.005–1.023]) while SBP was only associated with stroke (HR 95%CI 1.014[1.003–1.026]). On multivariate analysis, the highest PP quartile consistently associated with stroke and combined outcome (*p* < 0.05, all). This prospective cohort study suggesting that PP may be a pivotal predictor for CV risk.

## Introduction

Elevated blood pressure (BP) is the most important risk factor for cardiovascular morbidity and mortality both in China and worldwide^1,2^. In the Asia Pacific Cohort Studies Collaboration, BP is identified as a key determinant of the burden of stroke, ischemic heart disease, and overall cardiovascular mortality, with substantial potential benefits observed when systolic BP (SBP) is lowered to at least 115 mmHg^3^. A 5–6 mmHg SBP reduction reduces the risk of coronary artery disease (CAD) by 16% and stroke by 38%^4^. SBP is now increasingly recognized as more important parameter compared with diastolic BP (DBP), and robust investigations have focused on the appropriate target for SBP to reduce cardiovascular risk in specific populations^5^. Nevertheless, compared with SBP, the impact of pulse pressure (PP) on cardiovascular risk remains relatively less comprehensively elucidated up to now.

Prior studies have favored that PP constitutes a major risk factor for both cardiovascular and cerebrovascular events, particularly in hypertensive populations^6–8^. Meanwhile, some studies have shown that PP servers as a more potent predictor of cardiovascular events than SBP or DBP, especially among elderly patients^9^. To date, however, controversy exists as to the predictive power of PP in different populations. In patients with heart failure (HF), a meta-analysis of individual patient data from the MAGGIC collaboration, which included 22 studies and 27,046 patients—indicated that lower PP was an independent predictor of mortality in patients with HF and reduced ejection fraction (HF-REF). In contrast, this relationship was not consistently observed in patients with HF and preserved ejection fraction (HF-PEF)^10^. However, data from both PARAGON-HF trial^11^ and the TOPCAT trial^12^ consistently shown that high PP was an independent predictor of cardiovascular events in patients with HF-PEF. For patients with CAD, the notion was also inconsistent. The INVEST analysis involved 22,576 CAD patients with hypertension, and finally found that on the status of anti-hypertensive treatment, PP was a weaker predictor of cardiovascular outcomes than SBP, DBP, or mean arterial pressure (MAP)^13^. However, the analysis from the international REACH registry study enrolled stable outpatients with either established atherothrombotic disease (CAD, cerebrovascular disease, or peripheral artery disease) or with ≥3 risk factors for atherothrombosis. The data demonstrated that PP was associated with multiple adverse cardiovascular outcomes and provides prognostic utility beyond that of MAP^14^. Notably, wide PP has also been linked to other adverse vascular phenotypes, such as poor coronary collateralization in ST-elevation myocardial infarction^15^ and an increased risk of contrast-induced nephropathy^16^. Although the above investigations covered an impressive number of patients, the data have been generated from clinical trials with restricted enrolled patient populations about patients with CAD accompanied by hypertension (with relatively high baseline SBP and DBP) or patients with atherothrombosis (CAD patients only covered 58%, and 13.65% with HF). Furthermore, certain intervention was performed in clinical trial and could not reflect the standard secondary preventive therapy in patients with CAD. With regard to subjects with stable CAD and normal EF in an Asian population, there was no previous study systematically investigated its relationship to the coronary severity and adverse cardiovascular outcome so far.

Thus, the aim of this study is to investigate the clinical significance of PP with respect to coronary severity assessed by Gensini Score and with the cardiovascular outcomes in a cohort of large, Chinese populations with angiography-proven stable CAD and normal EF.

## Results

### Baseline characteristics

The baseline characteristics of the study participants were shown in Table 1. The mean age of the cohort was 57.6 ± 10.6 years, 32.7% were elder people, and 70.9% were male. The mean BMI was 25.88 ± 3.18 kg/m^2^ and the obesity covered 23.0%. A total of 3798 (54%) patients have smoking habits. Comorbidities were common, including hypertension (62.8%), hypercholesterolemia (75.2%), diabetes mellitus (DM) (27.7%), and history of stroke (3.4%). Baseline medication use were also detailed in Table 1.

**Table 1:** Clinical Characteristics of the Study Population

		Quartiles of PP (mm Hg)				
	All Cohort (*n* = 7027))	First Quartile (<40 mm Hg, *n* = 935)	Second Quartile (40–49 mm Hg, *n* = 2289)	Third Quartile (50–59 mm Hg, *n* = 2000)	Fourth Quartile (≥60 mm Hg, *n* = 1803)	*p* Value
Age, yrs	57.6 ± 10.6	52.5 ± 10.3	55.5 ± 10.3	57.9 ± 10.2	62.7 ± 9.3	<0.001
Elder, *n*(%)	2297 (32.7%)	138 (14.8%)	543 (23.7%)	658 (32.9%)	958 (53.1%)	<0.001
Male, *n*(%)	4980 (70.9%)	778 (83.2%)	1737 (75.9%)	1392 (69.6%)	1073 (59.5%)	<0.001
BMI, kg/m2	25.88 ± 3.18	26.02 ± 3.27	25.99 ± 3.31	25.87 ± 3.03	25.68 ± 3.14	0.011
Obesity, *n*(%)	1614 (23.0%)	238 (25.4%)	577 (25.2%)	438 (21.9%)	361 (20.0%)	<0.001
Heart Rate, bpm	69.8 ± 9.7	70.1 ± 10.3	69.5 ± 9.2	69.7 ± 9.3	69.9 ± 10.3	0.468
Creatine, ummol/L	78.1 ± 18.5	80.2 ± 18.5	78.8 ± 19.4	77.4 ± 18.1	76.7 ± 17.5	<0.001
SBP, mm Hg	127.1 ± 17.2	109.2 ± 11.5	118.5 ± 10.1	129.0 ± 10.6	145.3 ± 15.1	<0.001
DBP, mm Hg	77.9 ± 10.8	78.3 ± 11.2	77.5 ± 9.9	78.2 ± 10.3	78.0 ± 12.0	0.105
MAP, mm Hg	94.3 ± 11.6	88.6 ± 11.2	91.1 ± 9.9	95.1 ± 10.4	100.5 ± 12.3	<0.001
PP, mm Hg	49.2 ± 13.4	31.0 ± 4.0	41.0 ± 2.1	50.8 ± 2.0	67.3 ± 9.7	<0.001
*Medical history*						
Current smoker, *n*(%)	3798 (54.0%)	590 (63.1%)	1351 (59.0%)	1048 (52.4%)	809 (44.9%)	<0.001
History of hypertension, *n*(%)	4414 (62.8%)	452 (48.3%)	1264 (55.2%)	1262 (63.1%)	1436 (79.6%)	<0.001
Hypercholesterolemia, *n*(%)	5287 (75.2%)	691 (73.9%)	1694 (74.0%)	1556 (77.8%)	1346 (74.7%)	0.016
Diabetes Mellitus, *n*(%)	1947 (27.7%)	198 (21.2%)	573 (25.0%)	568 (28.4%)	608 (33.7%)	<0.001
History of stroke, *n*(%)	240 (3.4%)	23 (2.5%)	57 (2.5%)	68 (3.4%)	92 (5.1%)	<0.001
*Baseline medication*						
ACEI, *n*(%)	807 (11.5%)	110 (11.8%)	240 (10.5%)	226 (11.3%)	231 (12.8%)	0.287
ARB, *n*(%)	871 (12.4%)	109 (11.6%)	268 (11.7%)	218 (10.9%)	276 (15.3%)	0.004
Beta-blockers, *n*(%)	3331 (47.4%)	453 (48.4%)	1103 (48.2%)	960 (48.0%)	815 (45.2%)	0.351
CCB, *n*(%)	1330 (18.9%)	131 (14.0%)	412 (18.0%)	378 (18.9%)	409 (22.7%)	<0.001
Diuretic agents, *n*(%)	138 (2.0%)	34 (3.6%)	39 (1.7%)	40 (2.0%)	25 (1.4%)	0.010
Statins, *n*(%)	5320 (75.7%)	709 (75.8%)	1753 (76.6%)	1526 (76.3%)	1332 (73.9%)	0.276
Antiplatelet agents, *n*(%)	6000 (85.4%)	825 (88.2%)	1955 (85.4%)	1716 (85.8%)	1504 (83.4%)	0.040

Values are mean ± SD or *n* (%). *BMI* body mass index; *SBP* Systolic blood pressure; *DBP* Diastolic blood pressure; *MAP* Mean arterial pressure; *PP* pulse pressure; *ACEI* Angiotensin-converting enzyme inhibitors; *ARB* Angiotensin II receptor antagonists; *CCB* Calcium-channel blockers.

In the current study, BP was relatively well controlled (SBP 127.1 ± 17.2 mmHg; DBP 77.9 ± 10.8 mmHg; MAP 94.3 ± 11.6 mmHg; PP 49.2 ± 13.4 mmHg). When grouped PP into quartiles (Cutoff ranges for each of the PP quartiles were as follows: quartile 1, <40 mmHg, *n* = 935; quartile 2, 40 ≤ PP < 50 mmHg, *n* = 2289; quartile 3, 50 ≤ PP < 60 mmHg, *n* = 2000; and quartile 4, ≥60 mmHg, *n* = 1803), higher PP quartiles were associated with older age, female population, and were more likely to have hypertension, DM, and previous stroke history (*p* < 0.05, all).

### Association of PP and coronary severity

Indicated in Table 2, Gensini Score (GS) was divided into quartiles (First Quartile: <13.0, *n* = 1740; Second Quartile: 13.0–26.9, *n* = 1764; Third Quartile: 27.0–47.9, *n* = 1665; Fourth Quartile: ≥48, *n* = 1858). The mean values of PP, SBP, DBP, and MAP were slightly distinctive among groups (*p* < 0.05, all), while both PP and SBP were increased by the elevation of Gensini Score quartiles. Significantly, PP, SBP, and MAP were all related to the highest Gensini Score quartiles analyzed by Logistic regression analysis (*p* < 0.05, all).

**Table 2:** Association of pulse pressure and other blood pressure components with severity of coronary artery assessed by Gensini Score

	Quartiles of Gensini Score				
	First Quartile (<13.0, *n* = 1740)	Second Quartile (13.0–26.9, *n* = 1764)	Third Quartile (27.0-47.9, *n* = 1665)	Fourth Quartile (≥48, *n* = 1858)	*p* Value
PP, mmHg	48.0 ± 12.5	49.2 ± 13.1	49.2 ± 13.5	49.9 ± 13.7	0.001
SBP, mmHg	125.4 ± 16.5	127.8 ± 17.3	127.1 ± 16.9	127.9 ± 17.3	<0.001
DBP, mmHg	77.3 ± 10.5	78.6 ± 10.9	78.0 ± 10.8	78.0 ± 11.1	0.018
MAP, mmHg	93.4 ± 11.4	95.0 ± 11.9	94.4 ± 11.5	94.6 ± 11.8	0.001
	Quartiles of Gensini Score				
	First Quartile	Second Quartile	Third Quartile	Fourth Quartile	
		OR (95%CI)	OR (95%CI)	OR (95%CI)	*p* Value
PP, mmHg	Reference	1.007 (1.002–1.012)^a^	1.007 (1.001–1.012)^a^	1.011 (1.006-1.016)^a^	<0.05
SBP, mmHg	Reference	1.009 (1.004–1.013)^a^	1.006 (1.002–1.011)^a^	1.009 (1.005-1.013)^a^	<0.05
DBP, mmHg	Reference	1.011 (1.004–1.017)^a^	1.005 (0.999–1.012)	1.006 (0.999-1.012)	>0.05
MAP, mmHg	Reference	1.012 (1.006–1.018)^a^	1.008 (1.001–1.014)^a^	1.010 (1.004-1.016)^a^	<0.05

*SBP* Systolic blood pressure; *DBP* Diastolic blood pressure; *MAP* Mean arterial pressure; *PP* pulse pressure.^a^indicated statistical significance.

**Fig. 1:**
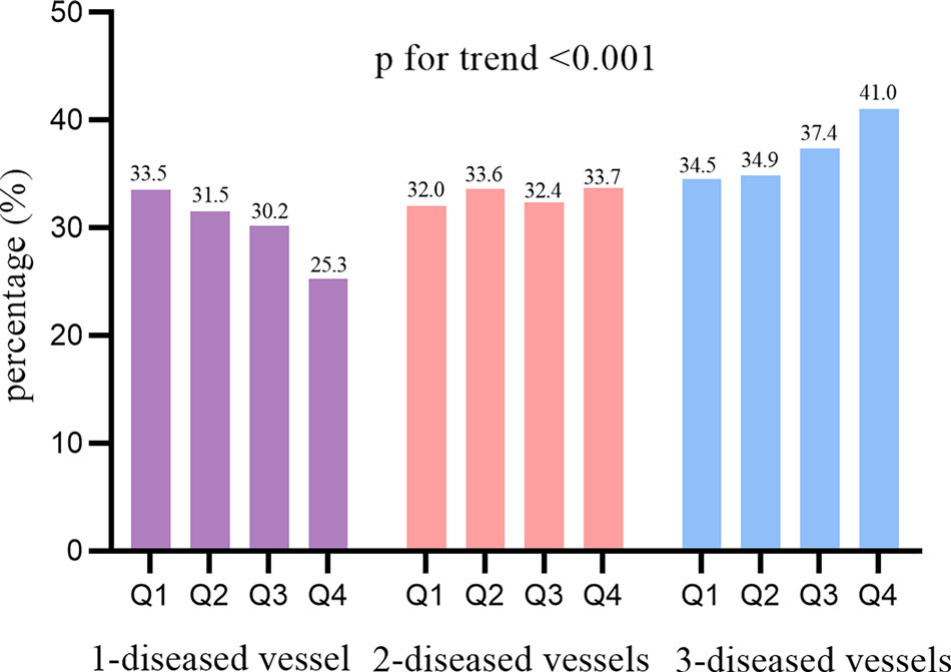
Pulse pressure quartiles and prevalence of diseased coronary vessels. This figure shows the linear trend in the prevalence of single-vessel, two-vessel, and three-vessel coronary disease across pulse pressure (PP) quartiles (Q1: <40 mmHg, Q2: 40–49 mmHg, Q3: 50–59 mmHg, Q4: ≥60 mmHg) in the study cohort. The percentage of patients with single-vessel disease decreased progressively from Q1 to Q4 (33.5% to 25.3%), while the percentage of patients with three-vessel disease increased significantly from Q1 to Q4 (34.5% to 41.0%); two-vessel disease prevalence showed no consistent trend. A test for linear trend confirmed a significant association between increasing PP quartiles and greater coronary vessel disease burden (*p* < 0.001). PP = pulse pressure.

Meanwhile, when divided PP into quartiles, the percentage of patients with 1-diseased vessel was decreasing (First Quartile vs. Second Quartile vs. Third Quartile vs. Fourth Quartile: 33.5% vs. 31.5% vs. 30.2% vs. 25.3%) while 3-diseased vessels was increasing (First Quartile vs. Second Quartile vs. Third Quartile vs. Fourth Quartile: 34.5% vs. 34.9% vs. 37.4% vs. 41.0%) with the increase of PP quartiles (*p* for trend<0.001, Fig. 1).

### Association of PP, SBP, DBP, and MAP with all cardiovascular outcomes

Over a median of 36.4 months (25th–75th percentile 22.3–53.5 months) follow-up period, 289 CVEs occurred (105 cardiovascular death, 62 non-fatal MI, and 122 strokes).

**Fig. 2:**
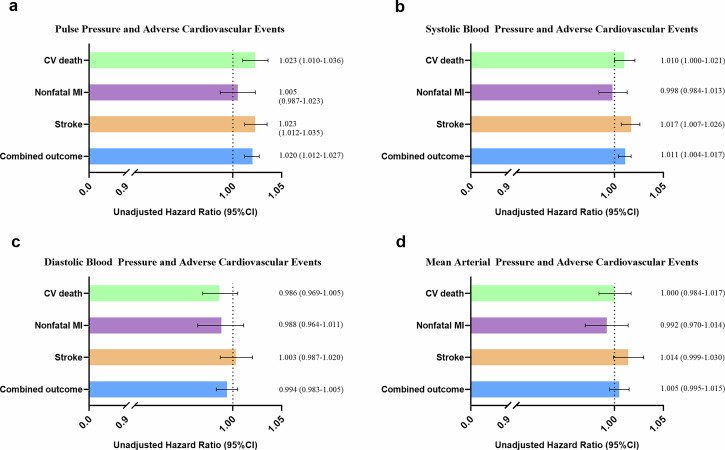
Unadjusted hazard ratios of blood pressure components for cardiovascular outcomes. This figure displays the unadjusted hazard ratios (HR) and 95% confidence intervals (CI) for each 10 mmHg increase in (**a**) pulse pressure (PP), (**b**) systolic blood pressure (SBP), (**c**) diastolic blood pressure (DBP), and (**d**) mean arterial pressure (MAP) for the study endpoints: cardiovascular (CV) death, nonfatal myocardial infarction (MI), stroke, and combined cardiovascular outcomes (CV death + nonfatal MI + stroke). PP was significantly associated with CV death, stroke, and combined outcomes (all *p* < 0.05); SBP was significantly associated with stroke and combined outcomes (all *p* < 0.05); DBP and MAP showed no significant associations with any of the cardiovascular endpoints (all *p* > 0.05). HR = hazard ratio; CI = confidence interval; PP = pulse pressure; SBP = systolic blood pressure; DBP = diastolic blood pressure; MAP = mean arterial pressure; CV = cardiovascular; MI = myocardial infarction.

Figure 2 indicated that PP was significantly associated with cardiovascular death (HR[95% CI]: 1.023 [1.010-1.036]), stroke (HR[95% CI]: 1.023 [1.012–1.035]), and the combined outcome (HR[95% CI]: 1.020 [1.012–1.027]). Similar results were also observed about SBP with stroke (HR[95% CI]: 1.017 [1.007–1.026]), and the combined outcome (HR[95% CI]: 1.011 [1.004–1.017]). Nevertheless, no significant correlation were found between DBP or MAP with any cardiovascular outcomes (*p* > 0.05, all).

**Fig. 3:**
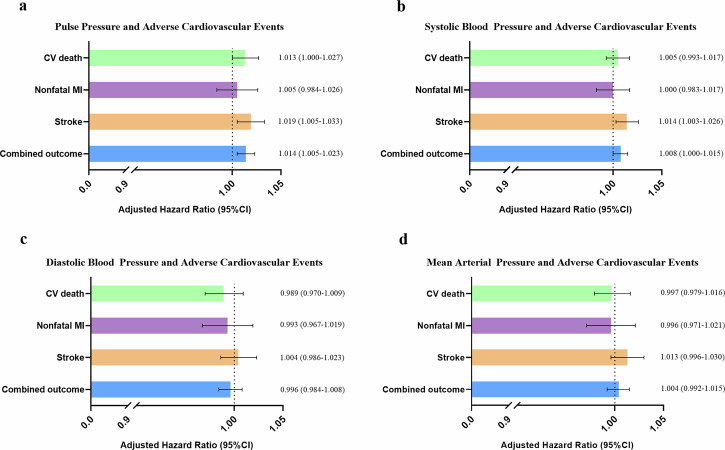
Adjusted hazard ratios of blood pressure components for cardiovascular outcomes. This figure presents the multivariable-adjusted hazard ratios (HR) and 95% confidence intervals (CI) for each 10 mmHg increase in (**a**) pulse pressure (PP), (**b**) systolic blood pressure (SBP), (**c**) diastolic blood pressure (DBP), and (**d**) mean arterial pressure (MAP) for cardiovascular (CV) death, nonfatal myocardial infarction (MI), stroke, and combined cardiovascular outcomes. Adjustments included sex, elderly status, obesity, smoking, hypercholesterolemia, diabetes mellitus, hypertension, heart rate, left ventricular ejection fraction, creatine, and antihypertensive agents use. PP remained independently associated with stroke and combined outcomes (all *p* < 0.05); SBP was only associated with stroke (*p* < 0.05); DBP and MAP showed no significant associations with any endpoint (all *p* > 0.05). HR = hazard ratio; CI = confidence interval; PP = pulse pressure; SBP = systolic blood pressure; DBP = diastolic blood pressure; MAP = mean arterial pressure; CV = cardiovascular; MI = myocardial infarction.

As shown in Fig. 3, in the next analysis adjusted for potential confounders including sex, elder, obesity, smoking, hypercholesterolemia, DM, hypertension, heart rate, left ventricular EF (LVEF), creatine, and antihypertensive agents, PP remains significantly associated with stroke (HR[95% CI]: 1.019 [1.005–1.033]), and combined outcome (HR[95% CI]: 1.014 [1.005–1.023]) while SBP was only associated with stroke (HR[95% CI]: 1.014 [1.003–1.026]).

### Association of categories of PP with all cardiovascular outcomes

**Fig. 4:**
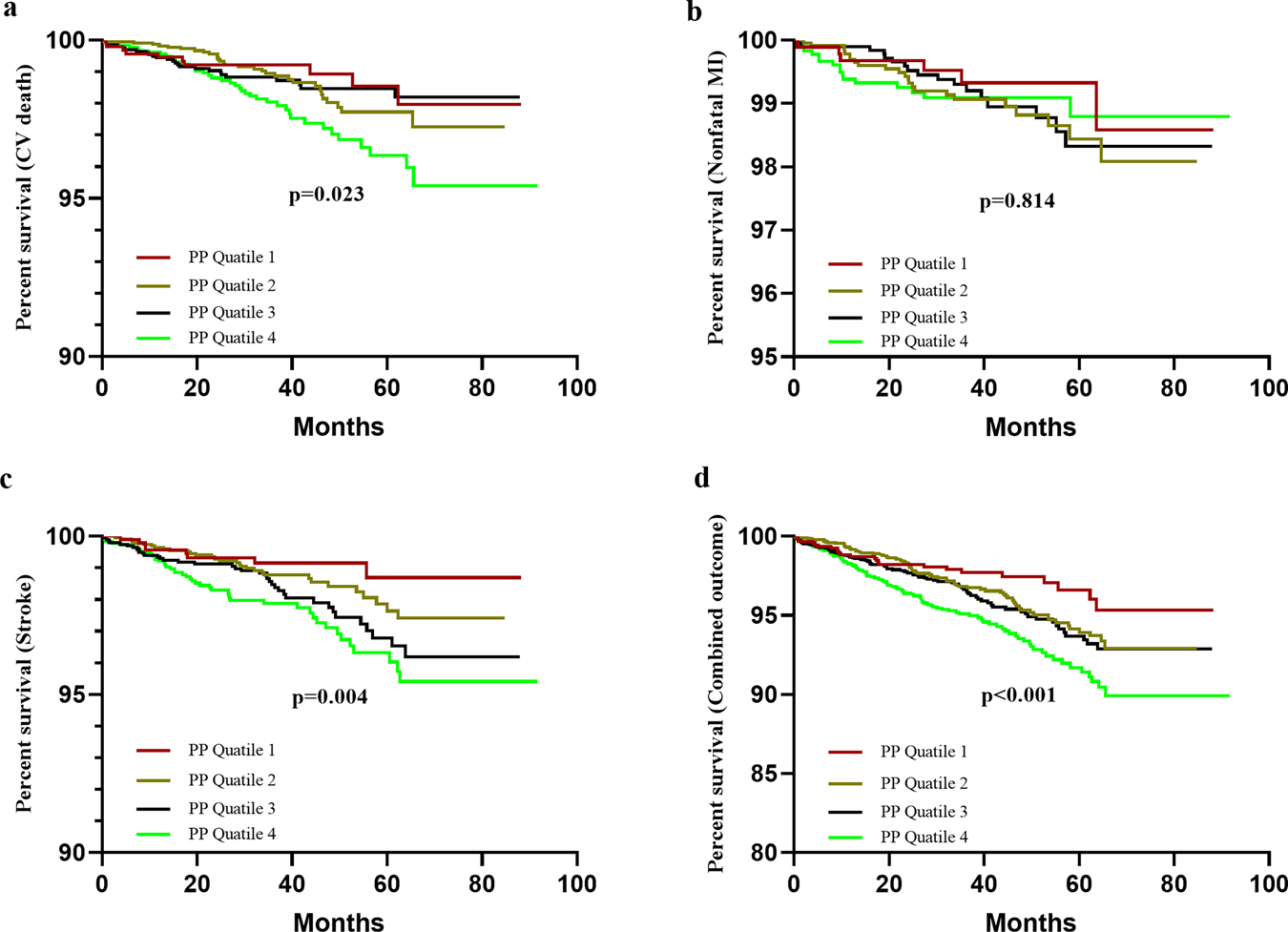
Kaplan-Meier survival curves for pulse pressure quartiles. This figure shows the Kaplan-Meier event-free survival curves for (**a**) cardiovascular (CV) death, (**b**) nonfatal myocardial infarction (MI), (**c**) stroke, and (**d**) combined cardiovascular outcomes across pulse pressure (PP) quartiles (Q1: <40 mmHg, Q2: 40–49 mmHg, Q3: 50–59 mmHg, Q4: ≥60 mmHg). The log-rank test confirmed the highest PP quartile (Q4) had the lowest event-free survival for CV death (*p* = 0.023), stroke (*p* = 0.004), and combined outcomes (*p* < 0.001). No significant difference in event-free survival for nonfatal MI was observed across PP quartiles (*p* = 0.814). PP = pulse pressure; CV = cardiovascular; MI = myocardial infarction.

Figure 4 displays the relationship of PP quartiles to all outcomes, including cardiovascular death, nonfatal myocardial infarction (MI), stroke, and the combined outcome. Kaplan-Meier analysis showed that the highest PP quartiles subjects had the lowest event-free survival rate among the four groups (cardiovascular death: *p* = 0.023; stroke: *p* = 0.004; and the combined outcome: *p* < 0.001) except for nonfatal MI (*p* = 0.814).

As presented in Table 3, univariate Cox regression models showed that the fourth PP quatiles had 2.119-fold (95% CI: 1.060-4.237, *p* = 0.034), 2.985-fold (95% CI: 1.407–6.333, *p* = 0.004), and 2.230-fold (95% CI: 1.429–3.480, *p* < 0.001) higher risk of cardiovascular death, stroke, and the combined outcome respectively compared with the first PP quatiles. Additional adjustment for other variables in the multivariate Cox regression models did not change the significance of the association with stroke (HR[95% CI]: 2.332 [1.008–5.396], *p* = 0.048) and the combined outcome (HR[95% CI]: 1.872 [1.117–3.140], *p* = 0.017; Table 4).

**Table 3:** Association of pulse pressure with adverse cardiovascular events on unadjusted analyses

		Unadjusted		
Outcome	Event Rate, n/N (%)	HR	95% CI	*p* Value
**CV death**	105/7027 (1.5%)			
Quartile 1	10/935 (1.1%)	Reference	Reference	Reference
Quartile 2	30/2289 (1.3%)	1.212	0.592–2.479	0.599
Quartile 3	25/2000 (1.3%)	1.172	0.563–2.439	0.672
Quartile 4	40/1803 (2.2%)	2.119	1.060–4.237	**0.034**
*Nonfatal MI*	62/7027 (0.9%)			
Quartile 1	6/935 (0.6%)	Reference	Reference	Reference
Quartile 2	23/2289 (1.0%)	1.542	0.628–3.786	0.345
Quartile 3	17/2000 (0.9%)	1.327	0.523–3.365	0.552
Quartile 4	16/1803 (0.9%)	1.410	0.552–3.604	0.473
*Stroke*	122/7027 (1.7%)			
Quartile 1	8/935 (0.9%)	Reference	Reference	Reference
Quartile 2	31/2289 (1.4%)	1.565	0.719v3.404	0.259
Quartile 3	38/2000 (1.9%)	2.231	1.041–4.782	**0.039**
Quartile 4	45/1803 (2.5%)	2.985	1.407–6.333	**0.004**
*Combined outcome*	289/7027 (4.1%)			
Quartile 1	24/935 (2.6%)	Reference	Reference	Reference
Quartile 2	84/2289 (3.7%)	1.412	0.897–2.223	0.136
Quartile 3	80/2000 (4.0%)	1.563	0.991-2.467	0.055
Quartile 4	101/1803 (5.6%)	2.230	1.429–3.480	**<0.001**

Univariate Cox regression analyses were performed. *CV* death, cardiovascular death; *MI* myocardial infarction.

*HR* hazard ration; *95%CI* 95% confidence interval.

**Table 4:** Association of pulse pressure with adverse cardiovascular events on multivariate-adjusted analyses

		Multivariate Adjusted		
Outcome	Event Rate, *n*/N (%)	HR	95% CI	*p* Value
*CV death*	105/7027 (1.5%)			
Quartile 1	10/935 (1.1%)	Reference	Reference	Reference
Quartile 2	30/2289 (1.3%)	1.400	0.610–3.214	0.427
Quartile 3	25/2000 (1.3%)	1.246	0.529–2.936	0.615
Quartile 4	40/1803 (2.2%)	1.690	0.728–3.924	0.222
*Nonfatal MI*	62/7027 (0.9%)			
Quartile 1	6/935 (0.6%)	Reference	Reference	Reference
Quartile 2	23/2289 (1.0%)	1.493	0.562–3.965	0.422
Quartile 3	17/2000 (0.9%)	1.244	0.448–3.454	0.675
Quartile 4	16/1803 (0.9%)	1.389	0.482–4.007	0.543
*Stroke*	122/7027 (1.7%)			
Quartile 1	8/935 (0.9%)	Reference	Reference	Reference
Quartile 2	31/2289 (1.4%)	1.343	0.584–3.090	0.487
Quartile 3	38/2000 (1.9%)	1.984	0.872–4.516	0.102
Quartile 4	45/1803 (2.5%)	2.332	1.008–5.396	**0.048**
*Combined outcome*	289/7027 (4.1%)			
Quartile 1	24/935 (2.6%)	Reference	Reference	Reference
Quartile 2	84/2289 (3.7%)	1.415	0.855–2.341	0.177
Quartile 3	80/2000 (4.0%)	1.525	0.914–2.543	0.106
Quartile 4	101/1803 (5.6%)	1.872	1.117–3.140	**0.017**

Multivariate Cox regression analyses were performed. The covariates including sex, elder, obesity, smoking, hypercholesterolemia, diabetes mellitus, hypertension, heart rate, LVEF, creatine, and antihypertensive agents. *CV death* cardiovascular death; *MI* myocardial infarction.

## Discussion

In this large, prospective cohort of Chinese patients with angiography-proven stable CAD and normal EF, we found that higher PP was significantly associated with greater coronary artery disease severity, assessed by both the Gensini Score and the prevalence of three-diseased vessels. More importantly, PP might emerge as an independent predictor of future stroke and composite cardiovascular events, even after adjusting for conventional risk factors and other BP components. In contrast, SBP lost its association with the composite outcome after adjustment, highlighting a potential distinct prognostic role for PP in this population.

PP, determined by both cardiac ejection and arterial stiffness, primarily reflects large artery compliance in individuals with normal cardiac function^17^. Our findings align with studies in HFpEF and the REACH registry, which support the prognostic value of high PP in patients with preserved EF or established atherosclerosis^11,12,14^. They contrast with the INVEST trial, which focused on treated hypertensive CAD patients, suggesting that the predictive power of PP may be context-dependent, influenced by baseline population characteristics, treatment intensity, and possibly blood pressure control levels^13^. Our study uniquely clarifies this relationship in a contemporary, real-world cohort of stable CAD patients with normal EF and relatively well-controlled BP, a population underrepresented in prior trials.

Till now, despite several studies on patients with CAD, there is still some debate as to the predictive value of PP in relating to cardiovascular risk in this special population. The INVEST analysis proved that PP was a weaker predictor of cardiovascular outcomes than SBP, DBP, or MAP^13^. On the contrast, the analysis from the international REACH registry study demonstrated that PP was associated with multiple adverse cardiovascular outcomes and provides prognostic utility beyond that of MAP^14^. However, the enrolled patients were not with CAD at stable status. The former study were CAD patients with hypertension, and the latter were patients with atherothrombosis. Additionally, the data have been generated from clinical trials which performed certain intervention in strictly selected people and could not reflect the standard secondary preventive therapy in the real-world. In the current study, the enrolled population were all suffered CAD defined by coronary angiography, with a predominantly hypertensive population and received standard secondary preventive therapy. It is worth noting that our study subjects were at stable status, with normal EF, and relatively well-controlled BP. Consequently, our data support the notion that baseline PP was a significant predictor of stroke and the combined outcome even after adjusting for multiple potential confounders.

Prior studies have evaluated the predictive power of PP in comparison with other BP components. Whether PP is superior to SBP and other indices for predicting cardiovascular outcomes remains in controversy. Some studies support PP is superior to SBP^7,18^, whereas others have shown SBP to be a stronger predictor of cardiovascular events than PP^6^. However, some studies were focused on the elderly people and some others not, hence, the participants age at the time of enrolment may be partly explain this diverge. In the current study, except for PP, we also examined the relationship of SBP, DBP and MAP with adverse cardiovascular outcomes. More importantly, our enrolled population were tended to be young (elder people only covered 32.7% of the whole, the mean age was 57.6years). The data finally shown that although PP and SBP followed the similar trends, in the multivariate Cox regression analysis SBP were only related to stroke but the relationship was not yielded for SBP, DBP, and MAP to combined outcome.

The stronger and more consistent association of PP with stroke, compared to MI, is physiologically plausible. Increased arterial stiffness and pulsatile stress, reflected by high PP, are more directly transmitted to the cerebral vasculature, contributing to microvascular damage and increasing the risk of cerebrovascular events^19,20^. This is further supported by a recent study linking wide PP to poor coronary collateralization, suggesting a broader impact on vascular adaptation and health^15^.

Our data suggested that PP, a valuable, easy-to-measure, low-cost biomarker, adds pivotal information in cardiovascular risk stratification. Additionally, clinicians should take PP into comprehensive decision-making with regard to the optimal BP management for those patients.

Our study has several limitations. First, the study subjects were all with CAD at stable status. Therefore, our results may not be generalizable to healthier or other specific populations. Second, the BP components were measured at baseline, and the alterations of these markers may also be clinically significant during the follow-up period. Third, the underlying mechanisms was not explored due to the observational design, and further investigations are needed in the near future.

In this prospective, large-sample cohort study of Chinese patients with angiography-proven stable CAD and normal EF, both PP and SBP were significantly associated with the coronary severity. Notably, PP was the independent predictor for stroke and combined outcomes after adjusting for potential confounders. Our data suggested that PP might add pivotal information in cardiovascular risk stratification. Hence, in real-world clinical practice, clinicians could take PP into comprehensive decision-making regarding the optimal SBP management for those patients.

## Methods

### Study population

The study complied with the Declaration of Helsinki and was approved by the hospital’s ethical review board (FuWai Hospital & National Center for Cardiovascular Diseases, Beijing, China). All enrolled subjects provided informed written consent in the current study.

From March 2011 to July 2017, a total of 14,316 Chinese patients hospitalized in our center were assessed in the current study. The inclusion criteria were patients with clinical symptoms such as angina pectoris, or chest distress. The exclusion criteria were as follows: (1) not performed angiography examination or coronary stenosis <50% in any coronary arteries; (2) acute coronary syndrome (ACS); (3) HF (both HF-REF and HF-PEF); (4) moderate-severe valvular diseases or myocardial disorders; (5) uncontrolled disease status such as severe liver and/or renal insufficiency, thyroid dysfunction, systematic inflammatory disease, hematologic disorders, and malignant disease; (6) without detailed BP data. Finally, a total of 7027 stable and angiography-proven CAD (coronary stenosis ≥50% of at least one coronary artery) patients were finally enrolled in the current analysis. All study patients were prescribed secondary prevention medicine of CAD and followed up for adverse outcomes.

### Follow-up

Patients were followed up at 6 months’ intervals through direct interviews or telephone by well-trained cardiologists or nurses who were blinded to the purpose of the study. The primary endpoints included cardiovascular death, non-fatal MI and stroke. For patients with suspected cardiovascular attacks, the medical records or emergency records were required to be sent to our centers. The endpoints were confirmed by at least two professional physicians.

### Definition of clinical status

Baseline height, weight, and seated SBP and DBP were obtained. Blood pressure was measured using a brachial mercury sphygmomanometer according to AHA/ACC guideline, and our previous study^21^. PP was defined as the difference between the SBP and DBP. A quality control check with the number of blood pressure readings ending in zero was performed. Body mass index (BMI) was calculated as weight (kg) divided by height (m) squared. Obesity was defined as patients with BMI ≥ 28 kg/m^2^. Current smokers were defined as having smoked a cigarette in the past 30 days and >100 cigarettes in a lifetime. The elder patients were defined as ≥60 years in female and ≥65 years in male.

Hypertension was defined as repeated systolic blood pressure ≥140 mmHg or diastolic blood pressure≥90 mmHg (at least two times in different environments) or currently taking anti-hypertensive drugs^22^. DM was diagnosed by fasting plasma glucose ≥7.0 mmol/L, the 2 h plasma glucose of the oral glucose tolerance test (OGTT) ≥ 11.1 mmol/L, or current use of hypoglycemic drugs or insulin^23^. Dyslipidemia was defined by medical history or fasting total cholesterol (TC) ≥ 5.18 mmol/L or triglyceride (TG) ≥ 1.7 mmol/L and/or high-density lipoprotein cholesterol (HDL-C) < 1.04 mmol/L (for male) or < 1.30 mmol/L (for female)^24^.

The diagnosis of Nonfatal MI included ST-segment–elevation MI (STEMI) and non–ST-segment–elevation MI (NSTEMI). STEMI was defined as elevated biomarkers and new or presumed new ST-segment elevation in 2 or more contiguous leads. NSTEMI was defined as the presence of elevated biomarkers and at least 1 of either ECG changes (ST-segment depression or T-wave abnormalities), or ischemic symptoms^25^. Stroke was diagnosed by the presence of typical symptoms and imaging.

### Severity of coronary atherosclerosis

The severity of coronary atherosclerosis was quantified by GS system^26^. It is a well-established, continuous quantitative tool for anatomical burden, and can more directly and sensitively reflect the total volume of atherosclerosis. Actually, there is other score systems in clinical practice, such as SYNTAX Score, which was aimed to evaluate the re-vascularization strategies. Therefore, we adopted GS system in this study.

The GS was computed by assigning a severity score to each coronary stenosis according to the degree of luminal narrowing and the importance of location, which was elaborate described in our previous study^27^.

### Statistical analysis

The data were expressed as the mean ± SD or median (Q1–Q3) for the continuous variables and the number (percentage) for the categorical variables. The differences among continuous variables were determined by the analysis of variance, and among the categorical variables were analyzed by χ2-test or Fisher’s exact test when appropriate. The event-free survival rates among groups were calculated by the Kaplan-Meier analysis and compared by the log-rank test. Univariate and multivariate Cox proportional hazard models were used to calculate the hazard ratio (HR) and 95% confidence interval (CI) to determine the relationship of PP (both continuous and categories), SBP (continuous), DBP (continuous), and MAP (continuous) with all outcomes. A p-values of less than 0.05 were considered statistically significant. The statistical analyses were performed with SPSS version 22.0 software (SPSS Inc., Chicago, IL, USA).

## Data Availability

The minimal dataset required to interpret, replicate, and build upon the study findings is available from the corresponding author upon reasonable request. The full raw dataset is not publicly available due to ethical restrictions related to protecting patient privacy and confidentiality.

## Code availability

No custom code or scripts were used for data generation or statistical analysis in this study. All analyses were performed using commercial SPSS software (version 22.0), with standard built-in statistical functions and no modified parameters/variables.
